# Clinically Usable Interleukin 12 Plasmid without an Antibiotic Resistance Gene: Functionality and Toxicity Study in Murine Melanoma Model

**DOI:** 10.3390/cancers10030060

**Published:** 2018-02-27

**Authors:** Urska Kamensek, Natasa Tesic, Gregor Sersa, Maja Cemazar

**Affiliations:** 1 Department of Experimental Oncology, Institute of Oncology Ljubljana, Zaloska 2, SI-1000 Ljubljana, Slovenia; ukamensek@onko-i.si (U.K.); gsersa@onko-i.si (G.S.); 2 Faculty of Health Sciences, University of Primorska, Polje 42, SI-6310 Isola, Slovenia; natasa.tesic@fvz.upr.si

**Keywords:** interleukin 12, antibiotic-free plasmid, gene electrotransfer, toxicity, expression, antitumor effectiveness, murine melanoma model

## Abstract

Plasmids, which are currently used in interleukin 12 (IL-12) gene electrotransfer (GET) clinical trials in the USA, contain antibiotic resistance genes and are thus, according to the safety recommendation of the European Medicines Agency (EMA), not suitable for clinical trials in the EU. In the current study, our aim was to prepare an IL-12 plasmid without an antibiotic resistance gene and test its functionality and toxicity after GET in a preclinical B16F10 mouse melanoma model. The antibiotic resistance-free plasmid encoding the human IL-12 fusion gene linked to the p21 promoter, i.e., p21-hIL-12-ORT, was constructed using operator-repressor titration (ORT) technology. Next, the expression profile of the plasmid after GET was determined in B16F10 cells and tumors. Additionally, blood chemistry, hematological and histological changes, and antitumor response were evaluated after GET of the plasmid in melanoma tumors. The results demonstrated a good expression and safety profile of the p21-hIL-12-ORT GET and indications of efficacy. We hope that the obtained results will help to accelerate the transfer of this promising treatment from preclinical studies to clinical application in the EU.

## 1. Introduction

Stimulating and exploiting the body’s own immune system to fight cancer is one of the most encouraging fields of cancer research today. Interleukin-12 (IL-12) is a key cytokine that regulates specific immune responses against cancer cells, and, as such, it holds great potential for cancer treatment [1]. Though proven an effective tumor suppressor in preclinical studies, clinical trials were held back by dose-limiting toxicity associated with its systemic administration [2,3], giving way to new approaches that support more localized delivery of this potent cytokine, such as gene therapy approaches [4], including gene electrotransfer (GET) [5]. 

At present, electrotransfer-mediated delivery of plasmids encoding IL-12 (IL-12 GET) is approaching clinical use for treatment of various superficial solid tumors. The main reason for the success of IL-12 GET is that it allows for the localized and prolonged steady production of IL-12, therefore overcoming two major obstacles the first clinical studies using recombinant IL-12 faced: e.g., toxicity at high doses and a fading effect of repeated administration [2,3]. Furthermore, compared to other gene therapy approaches using either ex vivo or in vivo gene transfer of IL-12 [4], IL-12 GET is relatively simple, economical, and safe method to achieve expression of IL-12 directly at the tumor site, where it can recruit the immune cells and activate them against the tumor antigens present there.

Five clinical trials were completed by the end of 2017 [6], all in the USA, in patients with malignant melanoma, cutaneous lymphoma, squamous cell carcinoma of the head and neck, and Merkel cell cancer (NCT00323206, NCT01502293, NCT01579318, NCT02345330, NCT01440816). Moreover, the development of IL-12 GET was put on the Fast track by the U.S. Food and Drug Administration (FDA) for the treatment of metastatic melanoma following disease progression on pembrolizumab or nivolumab. The FDA also recently granted orphan drug status to ImmunoPulse IL-12 (Oncosec, San Diego, CA, USA) for unresectable metastatic melanoma. Ongoing clinical studies evaluating IL-12 GET include a phase I monotherapy study in patients with triple negative breast cancer and a phase II study combining IL-12 GET with pembrolizumab in patients with stage III/IV melanoma progressing on pembrolizumab or nivolumab treatment [6]. 

Plasmids used in the past and ongoing preclinical and clinical studies mostly encode IL-12 under constitutive promoters, either viral, e.g., CMV (cytomegalovirus), or endogenous, e.g., the EF-1α (elongation factor 1α) promoter, and they also carry an antibiotic resistance gene [5]. In Europe, such plasmids with antibiotic resistance genes cannot enter clinical trials. Namely, according to the safety recommendation of the European Medicines Agency (EMA) [7], antibiotics should be avoided during production of plasmids for human use to circumvent possible allergic reactions in patients and the spread of antibiotic resistance traits to environmental and commensal microbes [8]. Additional reason for using antibiotic resistance-free plasmids is that antibiotic resistance genes can be responsible for lower transfection efficiency, transcriptional inactivation, and inflammatory reactions [8,9].

Hence, in our group we are striving to prepare plasmids without the antibiotic resistance genes using the operator-repressor titration (ORT^®^) technology [10]. Moreover, in order to achieve more tailored expression of the therapeutic genes, we are replacing the promoters based on the envisioned use of the specific plasmid [10]. Since the IL-12 therapy is often planned as an adjuvant to conventional therapies, such as radiotherapy or chemotherapy, we decided to place the IL-12 coding sequence under the transcriptional control of an inducible p21 (or cyclin dependent kinase inhibitor 1A (CDKN1A)) promoter that can be activated by the genotoxic stress induced by these therapies. The promoter of the p21 gene was utilized before to drive the expression of inducible nitric oxide synthase [11,12,13] and mouse IL-12 by our group [14]. These studies proved its usability for therapy, inducible transcriptional targeting, and specificity for tumor cells. 

In the present manuscript, we describe the construction of an antibiotic resistance-free plasmid encoding the human IL-12 fusion gene linked to the p21 promoter, which we named p21-hIL-12-ORT. The plasmid should be suitable for human clinical studies, but before it enters clinical evaluation, preclinical functionality and toxicity studies are needed, which was our aim in this study. The expression profile of the plasmid after GET was determined in B16F10 mouse melanoma cells and tumors. Additionally, blood chemistry, hematological and histological changes, and antitumor response were evaluated after GET of the plasmid in melanoma tumors. The results demonstrated a good expression and safety profile of the p21-hIL-12-ORT GET and indications of efficacy. 

## 2. Results

### 2.1. Confirmation of Successful Construction of the Plasmid

**Figure 1:**
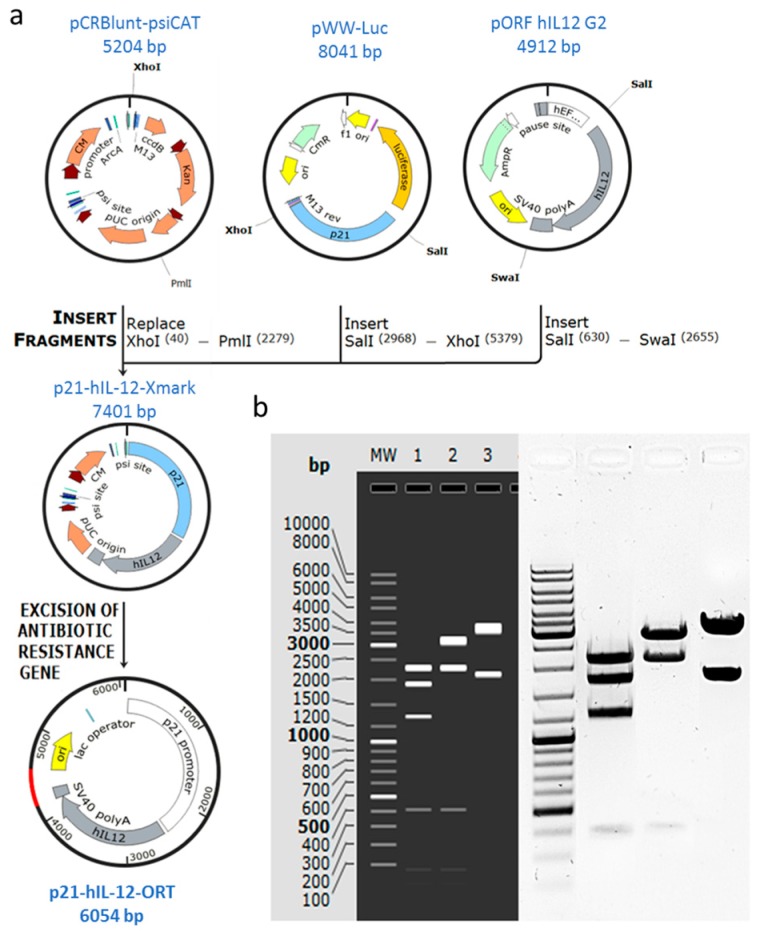
Construction and confirmation of the p21-hIL-12-ORT plasmid: (**a**) Schematic representation of the construction of the p21-hIL-12-ORT plasmid; (**b**) Restriction analysis results after electrophoresis on agarose gel confirming the identity, purity, and concentration of the plasmid. MW: GeneRuler DNAtm DNA Ladder Mix, lane 1: double restriction by *HindIII* and *MunI* resulting in 2338, 1925, 1338, 409, 79, and 19 bp bands; lane 2: restriction by *HindIII* resulting in 3260, 2338, 409, 79 bp, and 19 bp bands; lane 3: restriction by *KpnI* resulting in 3966 and 2142 bp bands.

The recombinant plasmid encoding hIL-12 under the transcriptional control of the p21 promoter and without the gene for antibiotic resistance was successfully constructed using standard molecular cloning methods and the antibiotic-free technology ORT^®^. Construction of p21-hIL-12-ORT was confirmed by restriction analysis (Figure 1) and sequencing. The plasmid was cut with different combinations of restriction enzymes and the identity of the plasmid was confirmed by positive matching of the pattern of bands on the electrophoresis gel to the expected pattern obtained by a simulation experiment using SnapGene software (1.1.3, GSL Biotech, Chicago, IL, USA) (Figure 1).

### 2.2. Expression Profile after GET In Vitro and In Vivo

**Figure 2:**
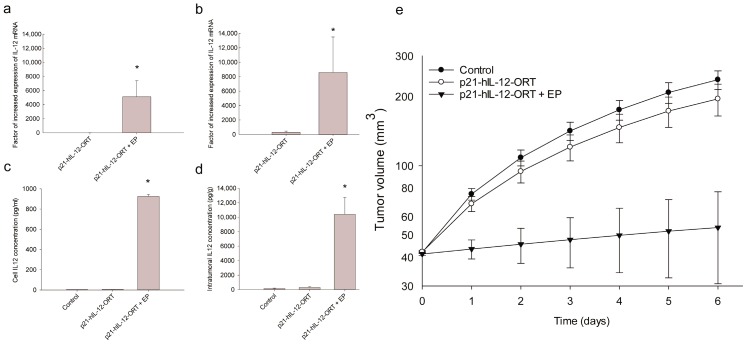
Expression of human interleukin 12 (hIL-12) and tumor growth after p21-hIL-12-ORT GET: Levels of hIL-12 mRNA 1 day after GET of p21-hIL-12-ORT in B16F10 cells (**a**) and in tumors (**b**) normalized to the mRNA levels in the control group; Concentration of hIL-12 protein 3 days after p21-hIL-12-ORT GET in cell media (**c**) and intratumorally (**d**); (**e**) Growth of B16F10 tumors after p21-hIL-12-ORT GET: intratumoral injection of H_2_O alone (Control), plasmid alone (p21-hIL-12-ORT), or in combination with application of EP (p21-hIL-12-ORT + EP). Data are presented as the mean with the standard error of the mean. In vitro experiments were repeated 3 times independently, and 6 animals per experimental group were included in the in vivo experiments. * *p* < 0.05 vs. Control and p21-hIL-12-ORT.

The expression of hIL-12 at the mRNA level in B16F10 cells and tumors one day after GET was significantly higher (Figure 2a,b) compared to control untreated cells and compared to cells treated with plasmid only, without electric pulses (EP). In vitro, the increase was by a factor of 4000–6000, and in vivo, the increase was even greater, by a factor of 8000–11,000. Furthermore, intratumoral and cell media concentrations of hIL-12 protein were also statistically significantly increased in B16F10 melanoma cells by a factor of 800–1000 and in tumors by a factor of 10,000–12,000, 3 days after p21-hIL-12-ORT GET (Figure 2b,c). 

### 2.3. Antitumor Effectiveness

After a single GET of p21-hIL-12-ORT in B16F10 melanoma tumors in vivo, reduction of tumor growth compared with all other groups was observed (Figure 2e), indicating that functional human IL-12 was expressed. Tumors from the experimental group that received only the plasmid injection, grew at the same growth rate as tumors from the untreated control group. 

### 2.4. Toxicity

Histological and hematological changes were evaluated at different time points after p21-hIL-12-ORT GET. Kidneys, lungs, and tumors were excised 6 and 8 days post-treatment for histological analysis, and blood was collected 1, 3, 6, and 8 days post-treatment. In all groups, the mice weight, as the measure of general condition, as well as tumor growth, was followed until the mice were sacrificed and autopsied. No significant fluctuations in weight were observed in any of the groups throughout the experiment, and no macroscopically visible metastases were noted during the autopsies.

#### 2.4.1. Histological Changes

**Figure 3:**
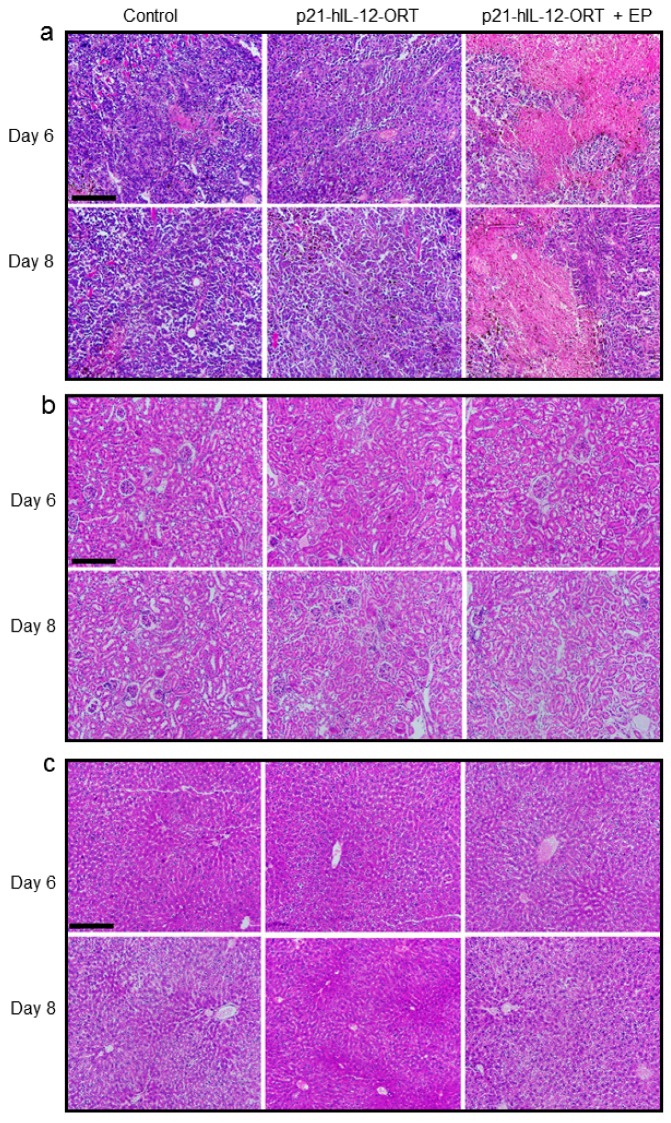
Histological changes in tumor kidney and lung tissue section, 6 and 8 days after p21-hIL-12-ORT GET in mouse B16F10 melanoma tumors. (**a**) Tumor necrosis in mouse B16F10 melanoma tumor sections; (**b**) Histological changes in kidney tissue; (**c**) Histological changes in lung tissue. Representative images of 2 µm thick paraffin embedded sections stained with hematoxylin and eosin. Images were taken at days 6 and 8 with a DP72 CCD camera (Olympus, Tokyo, Japan) connected to a Bx-51 microscope (Olympus). Scale bar 200 μm.

GET of p21-hIL-12-ORT caused pronounced necrosis of tumor tissue, which was observed 6 and 8 days post-treatment (Figure 3a). Central necrosis was also observed in tumor sections from the untreated control group and group receiving plasmid injection only, but to a much lesser extent. In kidney and lung tissue, 6 and 8 days after the p21-hIL-12-ORT GET, no abnormalities were noted (Figure 3b,c). 

#### 2.4.2. Blood Hematology

Minor differences in some standard hematology categories were observed in all experimental groups at all time points of blood collection post-treatment (Table 1 and Tables S1–S3). In mice from all experimental groups, erythrocytes (RBC) were slightly increased, while hemoglobin (HGB) was increased only in untreated mice at day 1 post-treatment. Furthermore, a significant decrease of RBC and HGB was observed in untreated mice and mice that received the plasmid injection only at day 8 post-treatment. Hematocrit (HCT) was increased in the mice from all experimental groups at days 1 and 3 post-treatment, while a significant HCT decrease was observed in untreated mice at day 8. Mice from all experimental groups showed lower than average mean corpuscular hemoglobin concentration (MCHC), mean cell hemoglobin concentration (CHCM), and cell hemoglobin (CH) at all time points post-treatment with no statistically significant differences between the groups. Red cell distribution (RDW) and hemoglobin distribution width (HDW) were increased but only at later time points. All other measured features were in the normal ranges. 

#### 2.4.3. Blood Chemistry

After GET, creatine and total serum protein values were not outside the limit of normal values (Table 2 and Tables S4 and S5). Albumin values in serum samples, taken from mice from all experimental groups on days 1, 6, and 8 post-treatment, were slightly higher than normal values. However, there were no statistically significant changes in albumin values between the experimental groups. 

## 3. Discussion

IL-12 GET is a very promising treatment for various superficial cancers, and it is currently under thorough clinical investigation in the USA using plasmids that contain antibiotic resistance genes. In the EU, however, such plasmids are not tolerated due to the safety concerns raised by the EMA regarding the presence of antibiotic resistance genes. In the current study, our aim was to prepare an IL-12 plasmid without an antibiotic resistance gene that would be suitable for clinical trials in the EU and to test its functionality and toxicity after GET in a preclinical B16F10 tumor model.

To prepare an antibiotic resistance-free plasmid, we used the ORT^®^ technology that was developed by Cobra Biologics (http://www.cobrabio.com/). The technology employs a plasmid-mediated repressor titration to activate a selectable marker in the host *E. coli* cells, removing the requirement for a plasmid-borne marker gene. The ORT technology was used before for the preparation of plasmids used for the DNA vaccination [15] and for the preparation of a plasmid encoding antiangiogenic metargidin peptide (AMEP) [16]. The latter plasmid was employed in three clinical trials (CT01764009, NCT01664273, NCT01045915) which examined the safety of GET [17]. The trails were performed in the EU and are so far the only GET clinical studies using antibiotic resistance-free plasmids for cancer treatment. The results showed that both intratumoral and intramuscular GET of AMEP are safe, but the trials were discontinued due to low enrolment rate and termination of the production of the plasmid DNA by the supplier.

In addition to removing the antibiotic resistance gene, we also replaced the constitutive promoter that is conventionally used to drive the expression of IL-12 [5], with an inducible and tumor specific p21 promoter. This is a native promoter of a gene that is present in all human cells [18] and is thus, in contrast to many viral promoters, not subjected to transcriptional inactivation by methylation, meaning it can provide longer lasting expression [14,19,20,21]. In our previous studies, we used this promoter to drive the expression of a reporter gene and proved it was inducible with therapeutic doses of radiation and cisplatin [14,20]. Since IL-12 GET was demonstrated to facilitate the induction of an immune response against tumor antigens [22,23], this therapy is particularly suitable as adjuvant to different ablative therapies where antigens are released from the killed cells, such as electrochemotherapy with cisplatin or radiotherapy [24]. The chosen p21 promoter should therefore be ideal for the combined treatments. This was confirmed in our earlier study, where we used the p21 promoter to drive the expression of a mouse IL-12 gene and GET was combined with tumor irradiation [14]. The treatment was performed in a TS/A tumor model and a good antitumor therapeutic effect was obtained, as compared to the same treatment using a constitutive promoter [14].

The antibiotic resistance-free plasmid encoding the human IL-12 gene under the p21 promoter, which we named p21-hIL-12-ORT, was tested for its expression after GET in B16F10 melanoma cells and tumors. On the mRNA level, factors of increase ranged from 4000 in vitro to 1100 in vivo; and on the protein level, from 800 in vitro to as far as 12,000 in vivo. These factors were larger compared to factors obtained in our previous study, where we used the mouse IL-12 under the p21 promoter [14]. The IL-12 plasmid used in the latter study still contained the antibiotic resistance gene, while in our new—human—IL-12 plasmid, the antibiotic resistance gene was removed. There are numerous studies demonstrating that the removal of the antibiotic resistance gene can have a positive effect on the expression profile [8,19]. Namely, antibiotic resistance genes are large prokaryotic genes that can hamper transfection efficiency due to the increased size of the plasmid DNA [25] and due to transcriptional inactivation of the promoter [26]. 

Antibiotic resistance genes can also induce the innate immune system and cause inflammatory reactions which are, in the case of localized delivery of immunostimulator genes such as IL-12, actually desirable. Thus, the removal of the antibiotic resistance gene could have a negative impact on the immunogenicity of the plasmid. However, one interesting observation in this and in our previous IL-12 GET studies [14] was that the measured IL-12 levels were much larger in vivo compared to in vitro. These higher in vivo levels could be the result of the induction of the immune response against the introduced foreign DNA, causing the production of endogenous IL-12 from the immune cells [27]. Thus, even though a large portion of the bacterial backbone was removed in the p21-hIL-12-ORT plasmid together with immunostimulatory sequences, it looks like we did not lose the adjuvant immunostimulatory effect of the plasmid DNA, possibly because a very large sequence for the p21 promoter was added, which can have a similar effect [28]. The described immunostimulatory effect of plasmid DNA is best demonstrated in studies testing electrotransfer of different control plasmid that showed that control plasmids, i.e., not carry any therapeutic gens, can lead to tumor growth delay in B16F10 tumor model [29,30,31]. Consequently, the antitumor effectiveness of electrotransferred therapeutic plasmids is a sum of the adjuvant effect of the plasmid vector itself and the expressed therapeutic gene, which is probably an additional reason for the success of IL-12 GET. 

In addition to expression, we also followed the therapeutic effectiveness and toxicity after in vivo intratumoral GET of the p21-hIL-12-ORT plasmid. There is no doubt that intratumoral IL-12 GET is an effective antitumor treatment [5,22,32], which was also confirmed in the current study. After a single GET of p21-hIL-12-ORT in B16F10 melanoma tumors, a significant reduction of tumor growth was observed. The treatment caused pronounced tumor necrosis observed on tumor tissue sections, 6 and 8 days after GET. On the other hand, there were no histopathological changes in kidney and lung tissue sections that would indicate systemic inflammation [33]. Likewise, the hematological tests demonstrated no significant toxicities associated with GET of the p21-hIL-12-ORT plasmid; moreover, the least abnormal values were observed in the therapeutic group, probably due to the reduced tumor burden in this group. Similar results were described in another preclinical GET study which evaluated the toxicities after GET of a mouse IL-12 in the B16F10 tumor model, and the treated mice were also found to be in the best health compared to control tumor-bearing mice [34]. 

Altogether, our results demonstrated a good expression and safety profile of p21-hIL-12-ORT GET and indications of efficacy. We hope that results obtained in this study will help to accelerate the transfer of this promising treatment from preclinical studies to clinical application in the EU.

## 4. Materials and Methods 

### 4.1. Construction of the p21-hIL-12-ORT Plasmid

The human IL-12 fusion gene originated from the commercially available pORF-hIL-12 G2 plasmid (InvivoGen, San Diego, CA, USA), and the coding sequence for p21 originated from the WWP-Luc plasmid from Bert Vogelstein’s lab (Addgene plasmid # 16451, Cambridge, MA, USA). The pCRBluntPsiCat X-mark plasmid, which was obtained from Cobra Biologics (Keele, UK), was used to prepare the antibiotic resistance-free plasmid. Plasmids were prepared by restriction endonuclease molecular cloning, which was followed by ligation and transformation into competent *E. coli* cells [35,36], using reagents (Miniprep kit, TransformAid Bacterial Transformation kit, *E. coli* strain JM107, restriction enzymes, Rapid DNA Ligation Kit, and GeneJET Gel Extraction Kit) from Thermo Fisher Scientific (Waltham, MA, USA). 

Two complementary technologies were used to prepare the antibiotic resistance-free plasmid: X-mark technology for the removal of the selectable marker gene and ORT technology for stable maintenance of the plasmid without the requirement for antibiotics. X-mark technology utilizes an *E. coli* strain DH1PEPA, which is a mutant for Xer recombination and enables stable replication of X-mark™ plasmids, in which antibiotic resistance gene (in our case chloramphenicol) is flanked by psi sites for Xer recombination. Transformation of X-mark™ plasmid into an ORT^®^ strain, or any other strain, results in excision of the antibiotic resistance gene by Xer recombination. ORT technology utilizes an *E. coli* stain DH1 ORT, in which essential bacterial chromosomal gene for an essential amino acid is controlled by an inducible lac promoter bound with the repressor protein, meaning that cells cannot grow. Transformation with a multi-copy plasmid also containing the lac operator causes the titration of the repressor from the chromosomal Lac promoter, leading to the expression of the essential amino acid and selective growth of the bacteria that contain the plasmids.

To prepare the expression cassette, the p21 sequence was cut out of WWW-luc plasmid by *SalI* and *XhoI* restriction enzymes, and the hIL12 sequence was cut out of the pORF hIL12 G2 plasmid by the *SalI* and *SwaI* restriction enzymes. Both sequences were ligated using the T4 DNA ligase into the pCRBlunt-psiCAT plasmid cut with *XhoI* and *PmlI* restriction enzymes. The resulting recombinant plasmid, p21-hIL-12-Xmark, still with the chloramphenicol resistance gene, was transformed into the DH1-PEPA *E. coli* cells (Cobra Biologics, Keele, UK) using the TranformAid Bacterial Transformation kit, and transformed clones were selected from the plates with LB agar growth media with added chloramphenicol (Merck, Kenilworth, NJ, USA). The p21-hIL-12-Xmark plasmid was than transformed into the DH1-ORT *E. coli* cells (Cobra Biologics) in which the antibiotic resistance gene was excised, resulting in the antibiotic resistance-free p21-hIL-12-ORT plasmid. The constructed plasmid was evaluated for stability by subculturing on agar plates without any added antibiotic and its nucleotide sequence was confirmed by restriction analysis. Additionally, the plasmid was sequenced using the complete plasmid sequencing service by MGH CCIB DNA Core (Boston, MA, USA).

### 4.2. Production and Purification of the Plasmid DNA

The p21-hIL-12-ORT plasmid was isolated and purified using an EndoFree PlasmidMega Kit (Qiagen, Hilden, Germany) according to the instructions provided with the kit. The plasmid DNA was eluted in endotoxin-free water (Qiagen) to a concentration of 1 mg/mL. The purity and yields were spectrophotometrically determined (Epoch microplate spectrophotometer, Take3™ micro-volume plate, BioTek, Bad Friedrichshall, Germany). Additionally, the concentration and identity were confirmed by restriction analysis on an electrophoretic gel.

### 4.3. Cells

The B16F10 murine melanoma cell line (American Type Culture Collection, Manassas, VA, USA) was cultured in an advanced minimum essential medium (AMEM, Gibco, Thermo Fisher Scientific, supplemented with 5% fetal bovine serum (FBS, Gibco), 10 mL/L l-glutamine (GlutaMAx, Gibco), 100 U/mL penicillin (Grünenthal, Aachen, Germany), and 50 µg/mL gentamicin (Gibco) in a 5% CO_2_ humidified incubator at 37 °C. B16F10 is a well-established and widely used model for human skin cancers, which was also used in the IL-12-GET toxicity study [34] prior to the initiation of the first clinical study in USA.

### 4.4. Animals and Tumors

Female C57Bl/6 mice, 6–8 weeks old, were purchased from Envigo (Udine, Italy). The mice were maintained in a specific pathogen-free colony at constant room temperature (21 °C) and a 12 h light/dark cycle. Water and food were provided ad libitum. All experiments were performed in accordance with the official guidelines of EU Directive 2010/63/EU, and with permission from the Veterinary Administration of the Ministry of Agriculture, Forestry and Food of the Republic of Slovenia (permission no. 34401-4/2012/4, April 2012). B16F10 tumors were induced by subcutaneous injection, in the right flank of the mice, of 1 × 10^6^ viable tumor cells, in 0.1 mL of 0.9% sodium chloride (NaCl), which were prepared from cell culture in vitro. When tumors reached 40 mm^3^ (i.e., 6 mm in the longest diameter) mice were randomly divided into different treatment groups and subjected to a specific experimental protocol.

### 4.5. In Vitro GET

Murine B16F10 melanoma cells were trypsinized and washed in AMEM with 5% FBS. After 5 min of centrifugation, the cell pellet was washed in ice-cold electroporation buffer (125 mM sucrose, 10 mM K_2_HPO_4_, 2.5 mM KH_2_PO_4_, 2 mM MgCl_2_) and resuspended at a concentration of 2.5 × 10^7^ cells/mL. The cells (1 × 10^6^) were then mixed with 10 µg of p21-hIL-12-ORT and placed between two parallel plate stainless steel electrodes with a 2 mm gap, connected to a pulse generator (Faculty of Electric Engineering, University of Ljubljana, Ljubljana, Slovenia) and subjected to 8 square-wave electric pulses (EP) at 100 V for a duration of 5 ms at a frequency of 1 Hz. Immediately after GET, 50 µL of FBS was added to the cells, which were incubated for 5 min at room temperature and then plated onto 10 cm^2^ Petri dishes (Greiner Bio-One, Frichenhausen, Germany) for further assays. 

### 4.6. In Vivo GET

In vivo GET was performed as described before [37,38]. When tumors reached a volume of 40 mm^3^, they were treated with intratumoral injection of 50 μg/50 μL [37] of the p21-hIL-12-ORT plasmid. After 10 min [38], tumors were placed between two parallel stainless steel electrodes with a 6 mm gap and EP were applied (8 square-wave EP of 240 V amplitude, 5 ms duration and a frequency of 1 Hz, given in two perpendicular directions). The EP were generated by an electric pulse generator Electro Cell B10 (Leroy biotech, Betatech, Saint-Orens-de-Gameville, France). The following groups were included in the experiment: Injection of H_2_O alone (Control), intratumoral injection of p21-hIL-12-ORT alone (p21-hIL-12-ORT) or in combination with EP (p21-hIL-12-ORT + EP). In each group, 5–6 mice were included. To comply with the 3R rule of animal experiments, additional group receiving a control plasmid instead of the therapeutic plasmid was not included in this study, because similar experiments were already performed in the scope of other studies demonstrating that GET of IL-12 results in better antitumor effectiveness than GET of control plasmids [34].

### 4.7. Reverse Transcription Polymerase Chain Reaction (RT-PCR)

For in vitro and in vivo determination of IL-12 mRNA levels, total RNA was isolated from the cells and tumors 1 day after p21-hIL-12-ORT GET using the TRIzol Plus RNA Purification System (Invitrogen, Thermo Fisher Scientific). Samples were collected from 3 independent in vitro experiments and from 6 animals per experimental group in vivo. One µg of total RNA was reverse transcribed into cDNA using SuperScript VILOTM cDNA Synthesis Kit (Invitrogen), according to the manufacturer’s instructions. Two μL of the 10-times and 100-times diluted mixture was used as the template for the RT-PCR using SYBR^®^ Green PCR Master Mix (Applied Biosystems, Thermo Fisher Scientific). A pair of primers was used to amplify hIL-12 and the housekeeping gene glyceraldehyde 3-phosphate dehydrogenase (GAPDH). RT-PCR was performed on a 7300 System (Applied Biosystems) as follows: activation of AmpliTaq Gold Enzyme (10 min at 95 °C), 45 cycles of denaturation (15 s at 95 °C), annealing and extension (1 min at 60 °C). The level of hIL-12 expression in each sample was calculated as the ratio of hIL-12 vs. reference gene GAPDH mRNA, and normalized to the untreated control group. 

### 4.8. Enzyme-Linked Immunosorbent Assay (ELISA)

To determine the protein concentration of IL-12, Quantikine^®^ ELISA Human IL-12 p70 Immunoassay (R&D Systems, Minneapolis, MN, USA) was performed according to the manufacturer’s instructions. For in vitro quantification, whole media from B16F10 melanoma cells was removed 3 days after p21-hIL-12-ORT GET, was centrifuged, and aliquots were stored at −80 °C for further processing. The experiments were repeated 3 times independently. For in vivo quantification, 6 mice per experimental group were sacrificed, and the tumors were removed, weighed and stored at −80 °C. Thereafter, the tumors were mechanically macerated in liquid nitrogen, and immediately after that, the tumor samples were resuspended in 500 μL of PBS containing protease inhibitors and centrifuged for 10 min at 10,000 rpm. The supernatant was separated from the sediment and stored at −80 °C until analysis. Concentrations of IL-12 were determined from the slope of a standard curve and calculated as pg of cytokine per mL of media or ng of cytokine per mg of tumor tissue. 

### 4.9. Blood Chemistry and Hematology

Blood samples were collected from the infraorbital sinus from 5 to 6 mice per experimental group 1, 3, 6, and 8 days post-treatment. Blood was stored at room temperature for approximately 2 h, and then hematology analysis was performed. For determination of chemistry changes, blood samples were stored for 20 min at 4 °C; serum was extracted by centrifugation at 3000 rpm for 5 min and stored at −20 °C until analysis. Blood and serum were analyzed at the Veterinary faculty Ljubljana using ADVIA 120 Hematology System (Siemens Healthineers, Erlangen, Germany) and compared with the established normal values. 

### 4.10. Antitumor Effectiveness

The antitumor effectiveness of gene p21-hIL-12-ORT GET was determined using the tumor growth delay assay. Tumors were measured in 3 perpendicular directions (a, b, c) using a digital caliper. Tumor volume was calculated by the formula: V  =  a  ×  b  ×  c  ×  π/6. Tumor growth was followed until the tumors reached an average volume of 200 mm^3^, after which the mice were sacrificed, autopsied, and examined for any macroscopically visible metastases. In all the treated mice, weight, as a measure of general condition, was followed.

### 4.11. Histology

On days 6 and 8 post treatment, 5–6 mice per experimental group were sacrificed and autopsied. During the autopsy, animals were macroscopically examined for metastasis and tissue samples of lung, kidney, spleen, heart, lymph nodes, and the skin above the tumor were excised. Samples were fixed in IHC zinc fixative (BD Pharmingen, Becton Dickinson Biosciences, Franklin Lakes, NJ, USA) for 24 h and then moved to 75% alcohol. Lung and kidney samples were selected for further histological analysis, because these tissues showed the most abnormal histology in a previous study [33]. These samples were embedded in paraffin, and tissue sections of 2 µm were cut from each paraffin block and stained with hematoxylin and eosin (HE). Images from each tissue slide were obtained under visible light at 20 × objective magnification, with a DP72 CCD camera (Olympus) connected to a BX-51 microscope (Olympus).

### 4.12. Statistical Analysis

Sigma Plot software (Systat software 13.0, London, UK) was used for statistical analysis. Statistical significance was determined by the Holm–Sidak method after one-way analysis of variance (ANOVA). Statistical significance was assumed at *p* < 0.05. The values are expressed as the arithmetic mean (AM) ± standard error of the mean (SEM).

## 5. Patents

Patent application WO/2017/137461 resulted from the work reported in this manuscript (International Application No: PCT/EP2017/052794).

## Figures and Tables

**Table 1:** Blood hematology 8 days after p21-hIL-12-ORT GET to B16F10 mouse melanoma tumors.

Test	Normals	Units	Day 8 (AM ± SE)		
			Ctrl	p21-hIL-12-ORT	p21-hIL-12-ORT + EP
WBC	3.2–12.7	×10^9^/L	6.1 ± 1.6	6.4 ± 1.4	6.2 ± 0.8
RBC	7.0–10.1	×10^12^/L	4.1 ± 1.0 *	5.4 ± 1.7	8.1 ± 1.2
HGB	118–149	g/L	59.6 ± 13.4 **	76.5 ± 26.4 +	121.0 ± 17.6
HCT	36.7–46.8	L/L	23.2 ± 4.8 ++	31.8 ± 8.7	46.3 ± 5.1
MCV	42.2–59.2	fL	57.5 ± 3.2	62.7 ± 5.0	60.5 ± 4.2
MCH	13.8–18.4	pg	14.6 ± 0.5	13.7 ± 1.5	14.9 ± 0.2
MCHC	310–347	g/L	256.4 ± 8.7	221.0 ± 26.1	252.1 ± 14.0
CHCM	307–340	g/L	238.0 ± 11.1	231.0 ± 11.3	230.8 ± 12.2
CH	13.8–18.4	pg	13.5 ± 0.3	14.0 ± 0.4	13.6 ± 0.2
RDW	11.7–15.1	%	17.0 ± 2.7	20.0 ± 2.6	16.7 ± 1.8
HDW	18–26	g/L	23.2 ± 2.1	27.3 ± 3.8	24.0 ± 2.7
PLT	766–1657	×10^9^/L	1001.4 ± 118.8	923.8 ± 87.7	1067.4 ± 101.8
MPV	5.0–8.0	fL	7.4 ± 0.4	7.6 ± 0.6	8.2 ± 0.8
% NEUT	6.8–31.1	%	27.1 ± 6.7	19.7 ± 5.4	9.5 ± 0.9
% LYMPH	60.2–95.0	%	67.7 ± 6.9	74.7 ± 5.3	83.0 ± 2.3
% MONO	0–4.3	%	2.1 ± 0.2	1.7 ± 0.3	2.2 ± 0.5
% EOS	0.2–5.9	%	1.7 ± 0.4	2.0 ± 0.7	4.3 ± 1.4
% BASO	0–1.0	%	0.2 ± 0.1	0.2 ± 0.1	0.2 ± 0.0
% LUC	0–3.2	%	1.3 ± 0.2	1.8 ± 0.9	0.8 ± 0.1
# NEUT	0.5–2.0	×10^9^/L	1.3 ± 0.2	1.2 ± 0.4	0.6 ± 0.1
# LYMPH	3.8–8.9	×10^9^/L	4.6 ± 1.4	5.0 ± 1.4	5.2 ± 0.8
# MONO	0–0.3	×10^9^/L	0.1 ± 0.0	0.1 ± 0.0	0.1 ± 0.0
# EOS	0–0.4	×10^9^/L	0.1 ± 0.0	0.1 ± 0.0	0.2 ± 0.1
# BASO	0–0.1	×10^9^/L	0.1 ± 0.0	0.1 ± 0.0	0.0 ± 0.0
# LUC	0–0.3	×10^9^/L	0.1 ± 0.0	0.1 ± 0.0	0.1 ± 0.0

WBC, White blood cell; RBC, Red blood cell; HGB, Hemoglobin; HTC, Hematocrit; MCV, Mean corpuscular volume (reflects the average volume of red cells); MCH, Mean corpuscular hemoglobin; MCHC, Mean corpuscular hemoglobin concentration; CHCM, Cell hemoglobin concentration mean; CH, cell hemoglobin; RDW, Red cell distribution width; HDW, Hemoglobin distribution width; PLT, Platelet count; MPV, Mean platelet volume; NEUT, Neutrophils; LYMPH, Lymphocytes; MONO, Monocytes; EOS, eosinophils; BASO, Basophils; LUC, Leukocytes; AM ± SE, arithmetic means ± standard error of the mean. * *p* < 0.05 compared to all groups at day 1; ** *p* < 0.05 compared to Control, p21-hIL-12-ORT + EP at day 1 and Control at day 3; + *p* < 0.05 compared to Control, p21-hIL-12-ORT+EP at day 1; ++ *p* < 0.05 compared to all groups at day 1, Control at day 3 and p21-hIL-12-ORT at day 6. #: number. 5–6 animals per experimental group.

**Table 2:** Blood chemistry 8 days after p21-hIL-12-ORT GET to B16F10 mouse melanoma tumors.

Test	Normals	Units	Day 8 (AM ± SE)		
			Ctrl	p21-hIL-12-ORT	p21-hIL-12-ORT + EP
Creatine	0.2–0.9	mg/dL	0.4 ± 0.0	0.4 ± 0.0	0.4 ± 0.0
TP2	3.5–7.2	g/dL	4.7 ± 0.2	4.2 ± 0.3	5.0 ± 0.3
Albumin	2.5–3	g/dL	**3.2 ± 0.3**	3.0 ± 0.2	**3.4 ± 0.2**

TP2, Total serum protein; AM ± SE, arithmetic means ± standard error of the mean. Abnormal values in bold. 5–6 animals per experimental group.

## Supplementary Materials

The following are available online at http://www.mdpi.com/2072-6694/10/3/60/s1, Table S1: Blood hematology 1 day after p21-hIL-12-ORT GET to B16F10 mouse melanoma tumors, Table S2: Blood chemistry changes 6 days after p21-hIL-12-ORT GET to B16F10 mouse melanoma tumors. Table S3: Blood hematology 6 days after p21–hIL–12–ORT GET to B16F10 mouse melanoma tumors. Table S4: Blood chemistry changes 1 day after p21-hIL-12-ORT GET to B16F10 mouse melanoma tumors. Table S5: Blood chemistry changes 6 days after p21-hIL-12-ORT GET to B16F10 mouse melanoma tumors.
