# Supplementary material for: Clinically Usable Interleukin 12 Plasmid without an Antibiotic Resistance Gene: Functionality and Toxicity Study in Murine Melanoma Model
Source: Cancers (Basel). 2018 Feb 27;10(3):60. doi: 10.3390/cancers10030060 (PMC5876635; doi:10.3390/cancers10030060)
Supplement: Supplementary file 1 [file cancers-10-00060-s001.pdf]

Supplementary Materials: Clinically Usable Interleukin 12 Plasmid without Antibiotic Resistance Gene: Functionality and Toxicity Study in Murine Melanoma Model

Urska Kamensek, Natasa Tesic, Gregor Sersa and Maja Cemazar

Table S1. Blood hematology 1 day after p21-hIL-12-ORT GET to B16F10 mouse melanoma tumors.

Test	Normals	Units	Day 1 (AM ± SE)		
			Ctrl	p21-hIL-12-ORT	p21-hIL-12-ORT+EP
WBC	3.2–12.7	×10⁹/L	4.7 ± 1.1	5.3 ± 0.7	3.7 ± 0.7
RBC	7.0–10.1	×10¹²/L	10.5 ± 0.1	10.4 ± 0.3	10.4 ± 0.1
HGB	118–149	g/L	155.2 ± 1.6	125.5 ± 28.4	153.7 ± 2.3
HCT	36.7–46.8	L/L	57.0 ± 1.3	55.8 ± 2.1	56.5 ± 0.9
MCV	42.2–59.2	fL	54.1 ± 0.8	53.2 ± 0.6	54.5 ± 0.4
MCH	13.8–18.4	pg	14.7 ± 0.1	12.0 ± 2.7	14.8 ± 0.1
MCHC	310–347	g/L	272.4 ± 3.4	224.8 ± 49.8	272.5 ± 1.8
CHCM	307–340	g/L	249.2 ± 4.6	255.8 ± 3.8	247.6 ± 1.3
CH	13.8–18.4	pg	13.4 ± 0.1	13.6 ± 0.1	13.5 ± 0.0
RDW	11.7–15.1	%	12.9 ± 0.3	12.9 ± 0.2	12.9 ± 0.1
HDW	18–26	g/L	18.2 ± 0.5	18.2 ± 0.3	18.4 ± 0.2
PLT	766–1657	×10⁹/L	1155.6 ± 86.4	986.0 ± 47.5	1106.5 ± 63.9
MPV	5.0–8.0	fL	7.2 ± 0.3	7.0 ± 0.3	7.5 ± 0.2
%NEUT	6.8–31.1	%	13.9 ± 1.7	11.0 ± 2.3	12.8 ± 1.5
%LYMPH	60.2–95.0	%	74.3 ± 4.1	82.8 ± 2.0	80.6 ± 1.3
%MONO	0–4.3	%	2.4 ± 0.6	1.3 ± 0.2	1.4 ± 0.3
%EOS	0.2–5.9	%	6.9 ± 2.5	3.3 ± 0.5	3.0 ± 0.6
%BASO	0–1.0	%	0.6 ± 0.2	0.3 ± 0.1	0.4 ± 0.1
%LUC	0–3.2	%	2.3 ± 0.9	1.4 ± 0.5	2.0 ± 0.6
#NEUT	0. 5–2.0	×10⁹/L	0.6 ± 0.1	0.5 ± 0.1	0.4 ± 0.1
#LYMPH	3.8–8.9	×10⁹/L	3.6 ± 1.0	4.4 ± 0.6	3.0 ± 0.6
#MONO	0–0.3	×10⁹/L	0.1 ± 0.0	0.1 ± 0.0	0.1 ± 0.0
#EOS	0–0.4	×10⁹/L	0.3 ± 0.1	0.2 ± 0.0	0.1 ± 0.0
#BASO	0–0.1	×10⁹/L	0.1 ± 0.0	0.0 ± 0.0	0.0 ± 0.0
#LUC	0–0.3	×10⁹/L	0.1 ± 0.0	0.1 ± 0.0	0.1 ± 0.0

WBC, White blood cell; RBC, Red blood cell; HGB, Hemoglobin; HTC, Hematocrit; MCV, Mean corpuscular volume (reflect average volume of red cells); MCH, Mean corpuscular hemoglobin; MCHC, Mean corpuscular hemoglobin concentration; CHCM, Cell hemoglobin concentration mean; CH, cell hemoglobin; RDW, Red cell distribution width; HDW, Hemoglobin distribution width; PLT, Platelet count; MPV, Mean platelet volume; NEUT, Neutrophils; LYMPH, Lymphocytes; MONO, Monocytes; eosinophils; BASO, Basophils; LUC, Leukocytes; AM ± SE, arithmetic means ± standard error of the mean. #: number. 5–6 animals per experimental group.

Table S2. Blood hematology 3 days after p21-hIL-12-ORT GET to B16F10 mouse melanoma tumors.

Test	Normals	Units	Day3(AM±SE)		
			Ctrl	p21–hIL–12–ORT	p21–hIL–12–ORT+EP
WBC	3.2–12.7	×10⁹/L	5.2 ± 0.3	4.9 ± 0.7	4.7 ± 0.6

RBC	7.0–10.1	$\times 10^{12}$/L	9.8 ± 0.7	8.9 ± 1.0	9.1 ± 1.2
HGB	118–149	g/L	145.3 ± 11.3	131.4 ± 15.3	132.4 ± 17.5
HCT	36.7–46.8	L/L	**53.0 ± 4.6**	**47.8 ± 5.6**	**48.8 ± 6.9**
MCV	42.2–59.2	fL	53.8 ± 0.9	53.5 ± 0.8	53.5 ± 0.9
MCH	13.8–18.4	pg	14.8 ± 0.1	14.7 ± 0.2	14.5 ± 0.2
MCHC	310–347	g/L	**274.3 ± 3.8**	**274.4 ± 0.8**	**270.8 ± 3.3**
CHCM	307–340	g/L	**250.0 ± 5.2**	**252.4 ± 3.2**	**248.6 ± 4.0**
CH	13.8–18.4	pg	**13.4 ± 0.1**	**13.4 ± 0.1**	**13.3 ± 0.1**
RDW	11.7–15.1	%	13.0 ± 0.3	13.4 ± 0.2	13.3 ± 0.5
HDW	18–26	g/L	18.6 ± 0.0	19.3 ± 0.4	19.8 ± 0.8
PLT	766–1657	$\times 10^{9}$/L	1055.8 ± 52.2	1017.8 ± 66.0	1319.8 ± 84.7
MPV	5.0–8.0	fL	6.9 ± 0.4	6.6 ± 0.4	7.2 ± 0.3
%NEUT	6.8–31.1	%	10.9 ± 2.2	11.1 ± 1.2	8.9 ± 1.2
%LYMPH	60.2–95.0	%	82.2 ± 2.7	81.7 ± 2.0	86.3 ± 1.7
%MONO	0–4.3	%	1.7 ± 0.3	1.6 ± 0.3	0.9 ± 0.2
%EOS	0.2–5.9	%	4.0 ± 1.1	4.5 ± 1.2	2.6 ± 0.6
%BASO	0–1.0	%	0.3 ± 0.1	0.2 ± 0.1	0.2 ± 0.1
%LUC	0–3.2	%	0.9 ± 0.2	0.9 ± 0.2	0.7 ± 0.1
#NEUT	0.5–2.0	$\times 10^{9}$/L	0.6 ± 0.1	0.5 ± 0.1	0.4 ± 0.1
#LYMPH	3.8–8.9	$\times 10^{9}$/L	4.3 ± 0.3	4.0 ± 0.6	4.1 ± 0.5
#MONO	0–0.3	$\times 10^{9}$/L	0.1 ± 0.0	0.1 ± 0.0	0.1 ± 0.0
#EOS	0–0.4	$\times 10^{9}$/L	0.2 ± 0.1	0.2 ± 0.1	0.1 ± 0.0
#BASO	0–0.1	$\times 10^{9}$/L	0.0 ± 0.0	0.0 ± 0.0	0.0 ± 0.0
#LUC	0–0.3	$\times 10^{9}$/L	0.0 ± 0.0	0.0 ± 0.0	0.0 ± 0.0

WBC, White blood cell; RBC, Red blood cell; HGB, Hemoglobin; HTC, Hematocrit; MCV, Mean corpuscular volume (reflect average volume of red cells); MCH, Mean corpuscular hemoglobin; MCHC, Mean corpuscular hemoglobin concentration; CHCM, Cell hemoglobin concentration mean; CH, cell hemoglobin; RDW, Red cell distribution width; HDW, Hemoglobin distribution width; PLT, Platelet count; MPV, Mean platelet volume; NEUT, Neutrophils; LYMPH, Lymphocytes; MONO, Monocytes; eosinophils; BASO, Basophils; LUC, Leukocytes; AM ± SE, arithmetic means ± standard error of the mean. #: number. Abnormal values in bold. 5–6 animals per experimental group.

Table S3. Blood hematology 6 days after p21–hIL–12–ORT GET to B16F10 mouse melanoma tumors. .

Test	Normals	Units	Day 6 (AM ± SE)		
			Ctrl	p21–hIL–12–ORT	p21–hIL–12–ORT+EP
WBC	3.2–12. 7	$\times 10^{9}$/L	7. 7 ± 0. 7	7. 0 ± 0. 2	6. 5 ± 0. 7
RBC	7.0–10.1	$\times 10^{12}$/L	**6. 3 ± 1. 3**	8. 8 ± 1. 2	9. 7 ± 0.5
HGB	118–149	g/L	**93. 3 ± 18. 4**	128. 8 ± 18. 1	140. 2 ± 8. 1
HCT	36.7–46.8	L/L	**35. 3 ± 5. 6**	46. 1 ± 5. 8	51. 6 ± 3. 1
MCV	42.2–59.2	fL	55. 5 ± 2. 8	53. 3 ± 1. 6	54. 5 ± 0. 7
MCH	13.8–18.4	pg	14. 9 ± 0. 3	14. 6 ± 0. 1	14. 8 ± 0.1
MCHC	310–347	g/L	**269.8 ± 8.8**	**275.3 ± 9.3**	**269.2 ± 4.3**
CHCM	307–340	g/L	**249.0 ± 10.3**	**255.6 ± 8.3**	**246.2 ± 3.1**
CH	13.8–18.4	pg	13.6 ± 0.2	**13.5 ± 0.1**	**13.4 ± 0.1**
RDW	11.7–15.1	%	**15.8 ± 1.8**	14.0 ± 1.3	14.9 ± 1.1
HDW	18–26	g/L	24.0 ± 3.7	19.3 ± 0.8	19.8 ± 0.9
PLT	766–1657	$\times 10^{9}$/L	995.3 ± 69.4	1126.0 ± 54.0	1284.8 ± 43.5
MPV	5.0–8.0	fL	7.2 ± 0.6	7.2 ± 0.4	7.5 ± 0.4
%NEUT	6.8–31.1	%	14.5 ± 1.7	11.7 ± 1.4	10.1 ± 1.1
%LYMPH	60.2–95.0	%	79.5 ± 1.6	81.2 ± 2.2	83.4 ± 1.7

%MONO	0–4.3	%	2.2 ± 0.4	2.2 ± 0.2	2.0 ± 0.3
%EOS	0.2–5.9	%	1.8 ± 0.2	3.5 ± 1.1	3.3 ± 0.8
%BASO	0–1.0	%	0.2 ± 0.0	0.2 ± 0.0	0.3 ± 0.0
%LUC	0–3.2	%	1.6 ± 0.1	1.3 ± 0.1	0.9 ± 0.1
#NEUT	0.5–2.0	×10^9/L	1.1 ± 0.2	0.8 ± 0.1	0.6 ± 0.0
#LYMPH	3.8–8.9	×10^9/L	6.1 ± 0.5	5.7 ± 0.3	5.4 ± 0.7
#MONO	0–0.3	×10^9/L	0.2 ± 0.0	0.2 ± 0.0	0.1 ± 0.0
#EOS	0–0.4	×10^9/L	0.1 ± 0.0	0.2 ± 0.1	0.2 ± 0.0
#BASO	0–0.1	×10^9/L	0.0 ± 0.0	0.0 ± 0.0	0.0 ± 0.0
#LUC	0–0.3	×10^9/L	0.0 ± 0.0	0.1 ± 0.0	0.1 ± 0.0

WBC, White blood cell; RBC, Red blood cell; HGB, Hemoglobin; HTC, Hematocrit; MCV, Mean corpuscular volume (reflect average volume of red cells); MCH, Mean corpuscular hemoglobin; MCHC, Mean corpuscular hemoglobin concentration; CHCM, Cell hemoglobin concentration mean; CH, cell hemoglobin; RDW, Red cell distribution width; HDW, Hemoglobin distribution width; PLT, Platelet count; MPV, Mean platelet volume; NEUT, Neutrophils; LYMPH, Lymphocytes; MONO, Monocytes; eosinophils; BASO, Basophils; LUC, Leukocytes; AM ± SE, arithmetic means ± standard error of the mean. #: number. Abnormal values in bold. 5–6 animals per experimental group.

Table S4. Blood chemistry changes 1 day after p21-hIL-12-ORT GET to B16F10 mouse melanoma tumors.

Test	Normals	Units	Day1(AM±SE)		
			Ctrl	p21–hIL–12–ORT	p21–hIL–12–ORT+EP
Creatine	0.2–0.9	mg/dl	0.4 ± 0.0	0.4 ± 0.0	0.4 ± 0.0
TP2	3.5–7.2	g/dl	5.3 ± 0.0	5.1 ± 0.1	5.4 ± 0.0
Albumin	2.5–3	g/dl	**3.6 ± 0.0**	**3.4 ± 0.2**	**3.6 ± 0.1**

TP2, Total serum protein; AM ± SE, arithmetic means ± standard error of the mean. Abnormal values in bold. 5–6 animals per experimental group.

Table S5. Blood chemistry changes 6 days after p21-hIL-12-ORT GET to B16F10 mouse melanoma tumors.

Test	Normals	Units	Day6(AM±SE)		
			Ctrl	p21–hIL–12–ORT	p21–hIL–12–ORT+EP
Creatine	0.2–0.9	mg/dl	0.4 ± 0.0	0.4 ± 0.0	0.4 ± 0.0
TP2	3.5–7.2	g/dl	4.5 ± 0.2	5.0 ± 0.3	5.2 ± 0.1
Albumin	2.5–3	g/dl	3.0 ± 0.1	**3.4 ± 0.2**	**3.5 ± 0.1**

TP2, Total serum protein; AM ± SE, arithmetic means ± standard error of the mean. Abnormal values in bold. 5–6 animals per experimental group.
